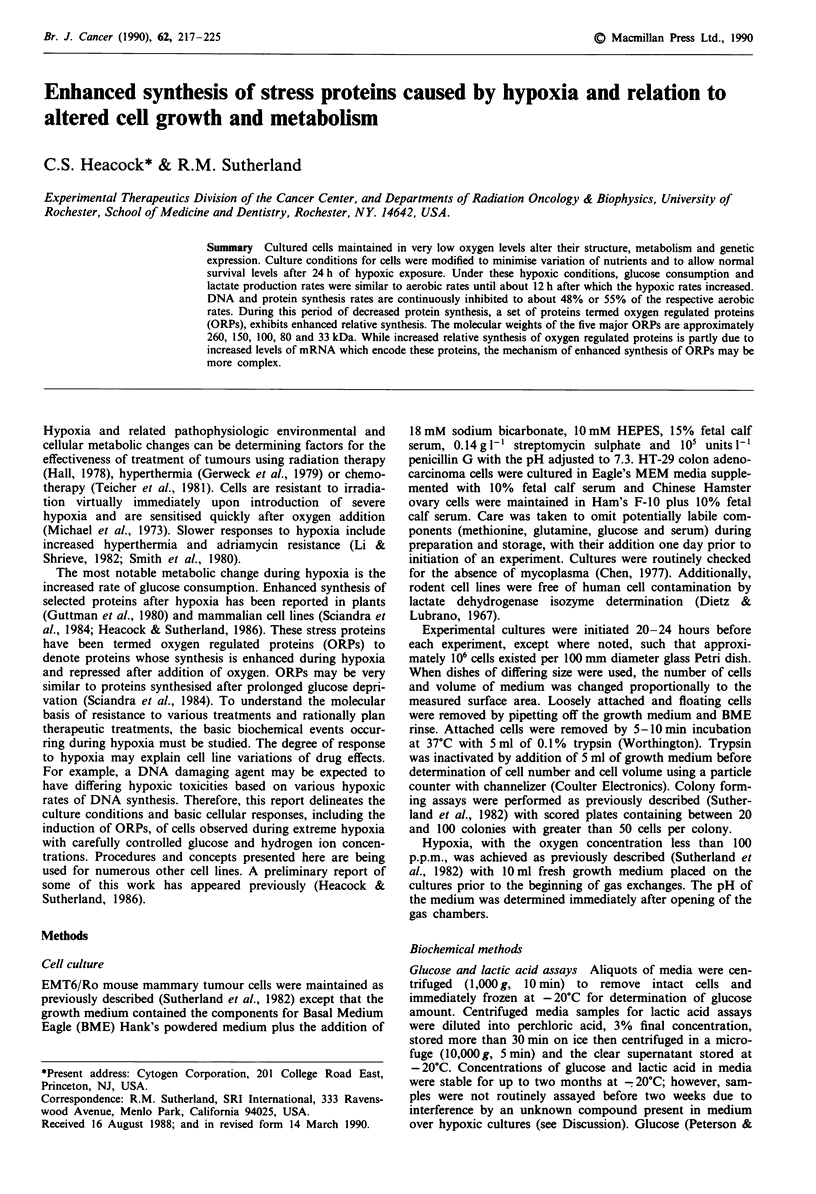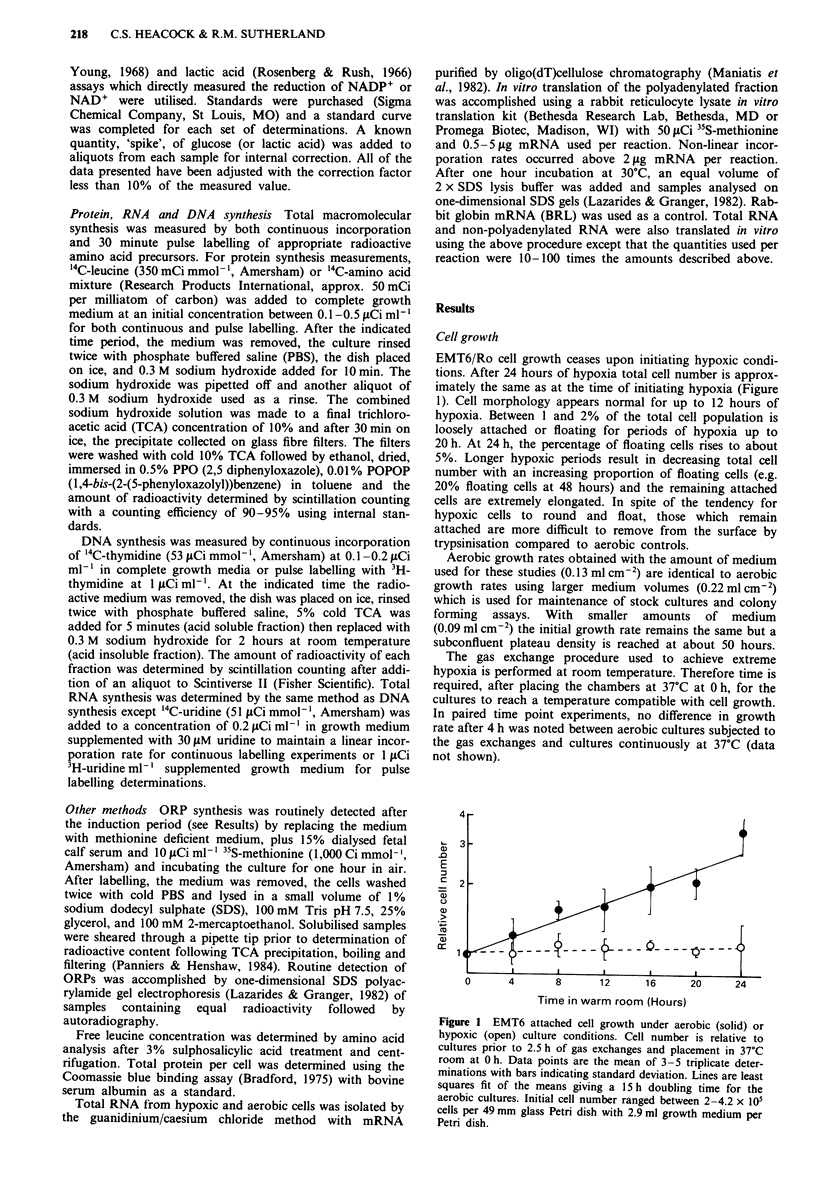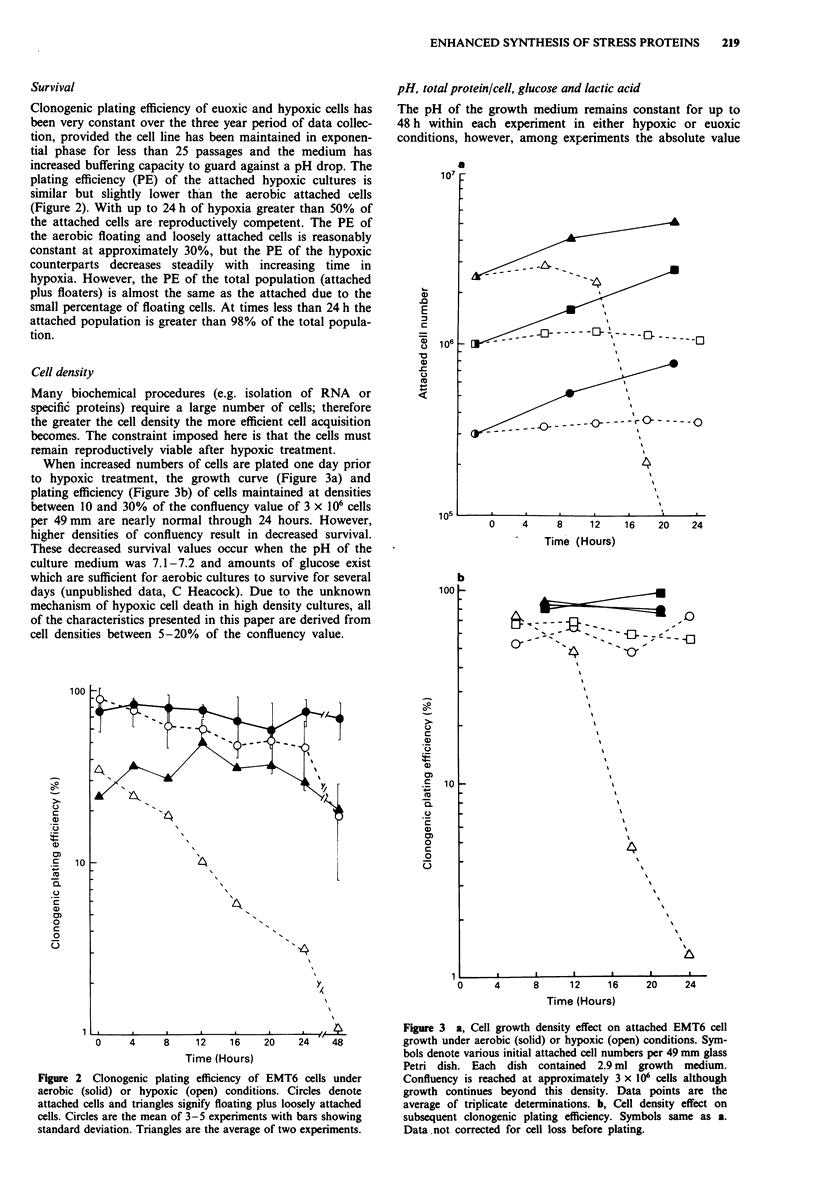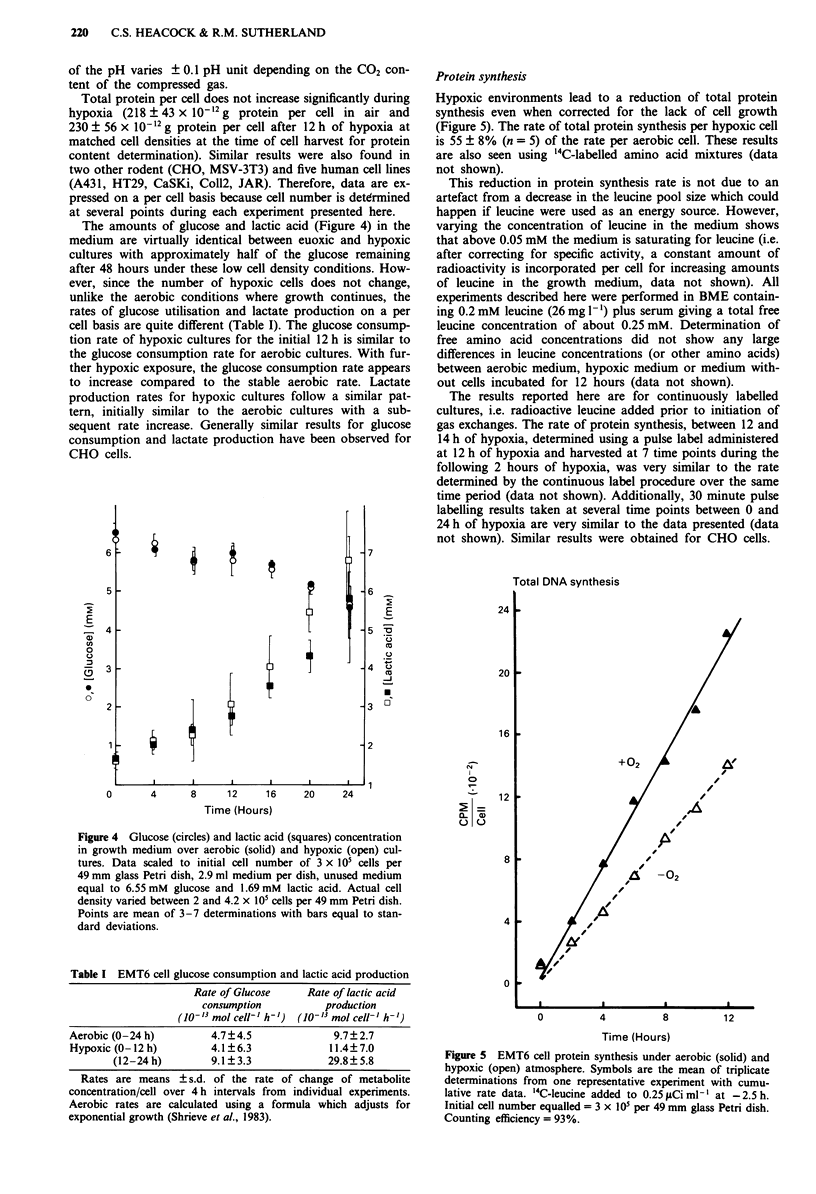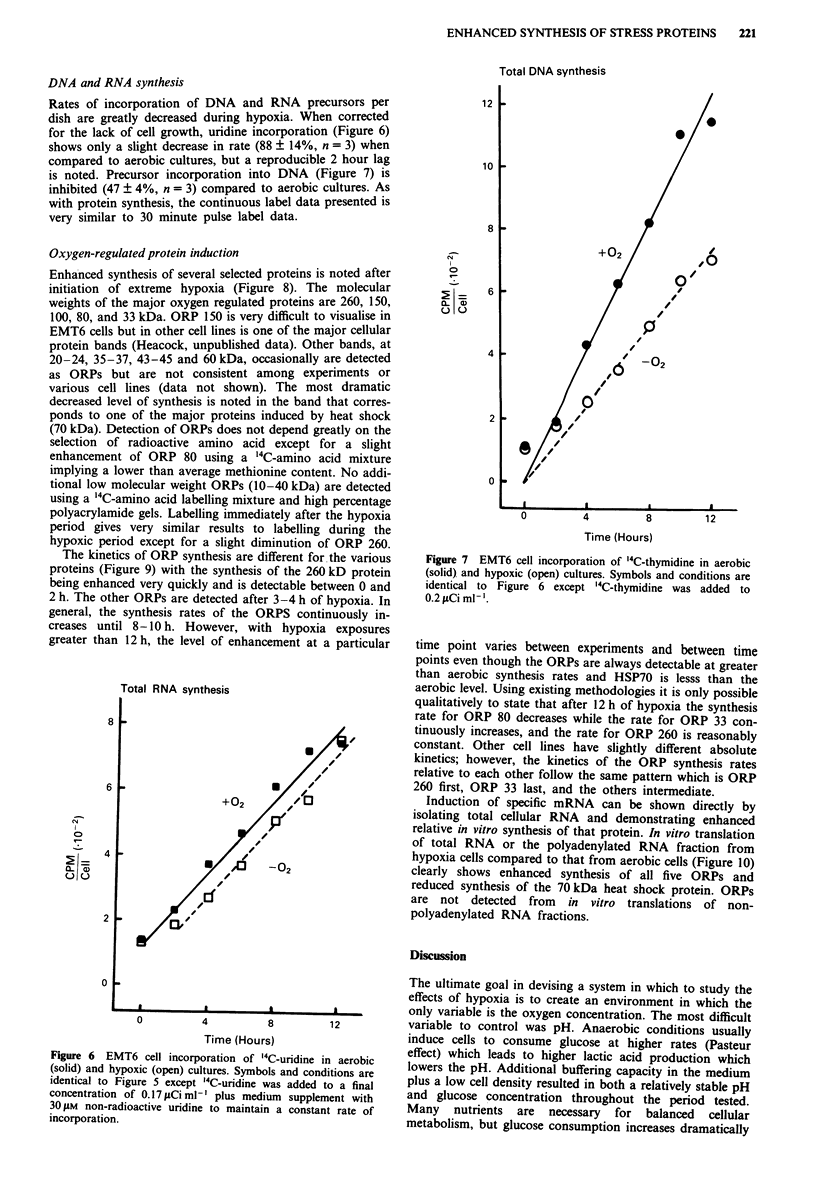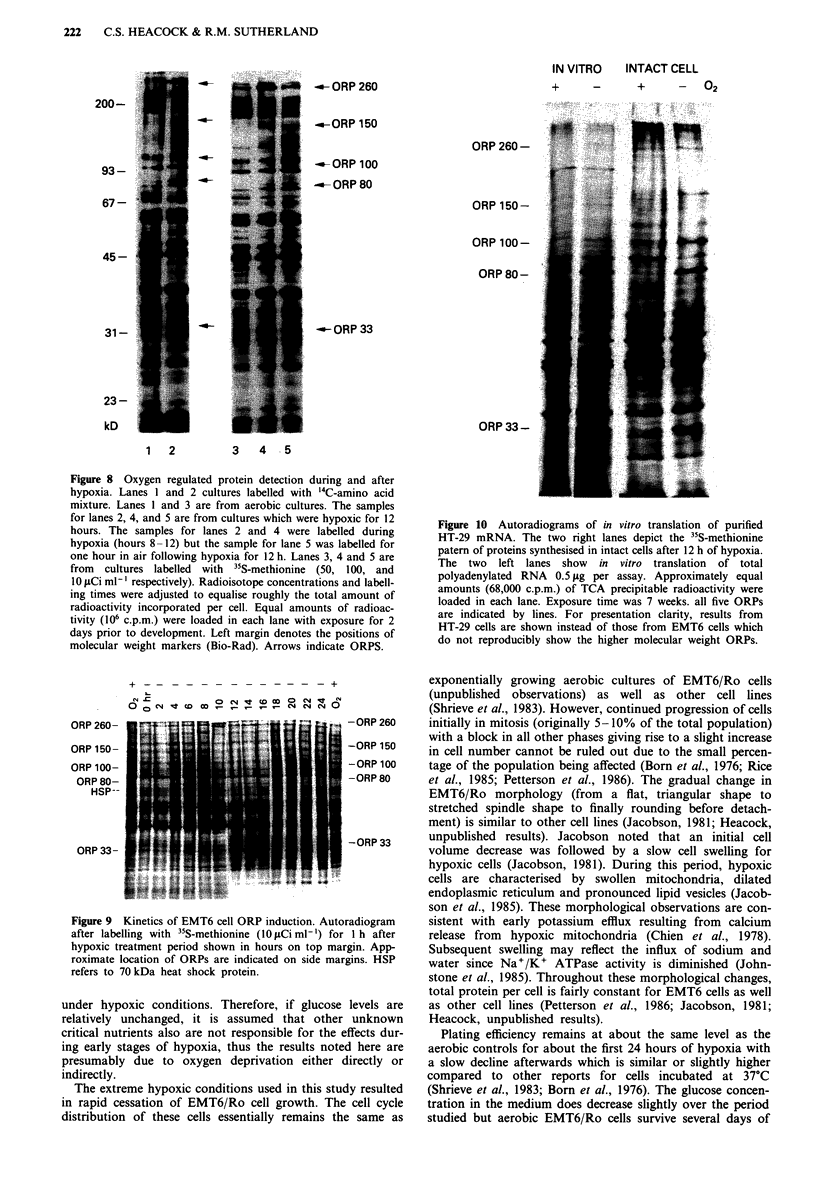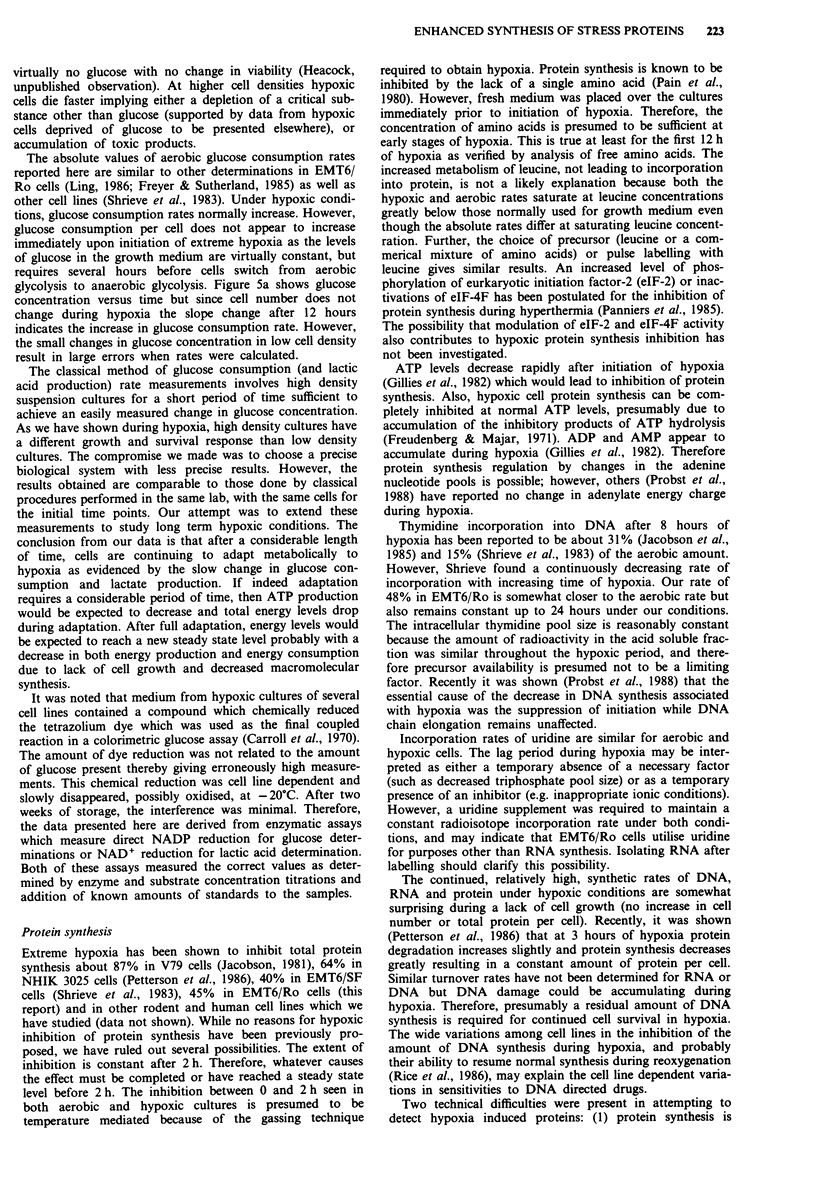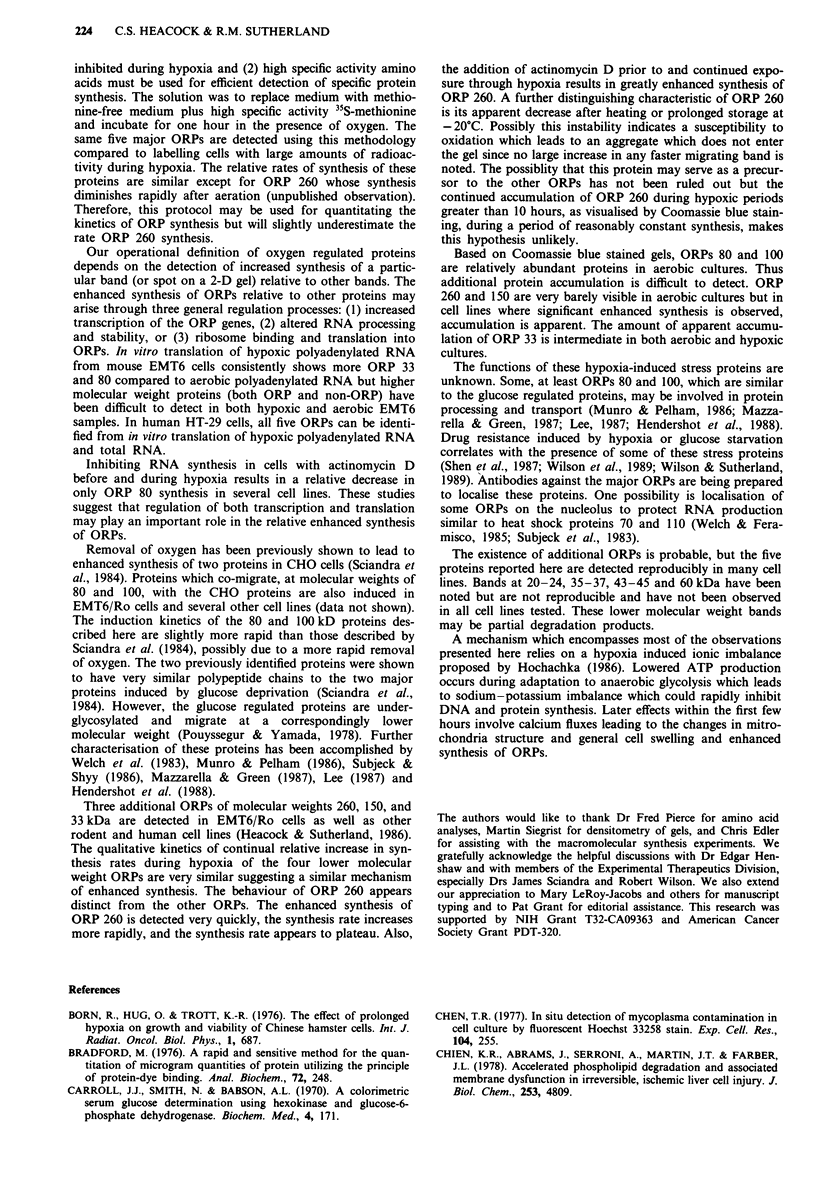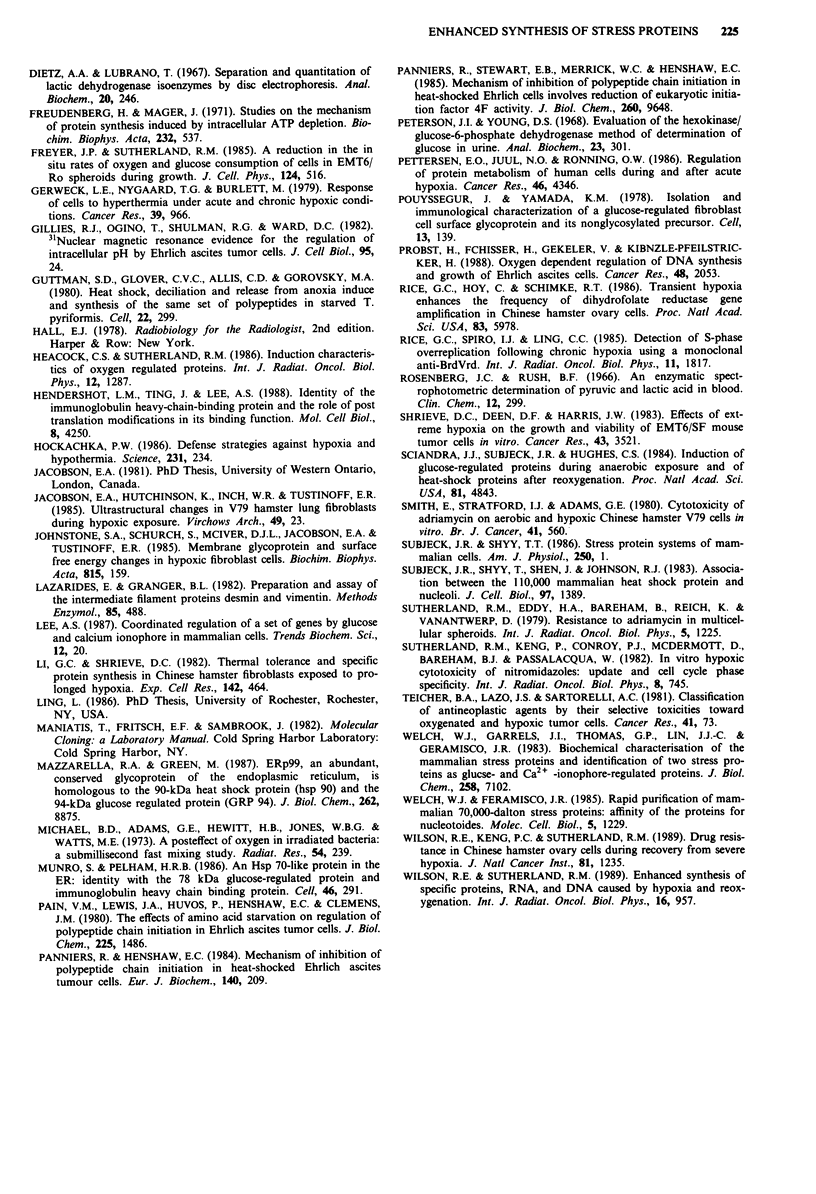# Enhanced synthesis of stress proteins caused by hypoxia and relation to altered cell growth and metabolism.

**DOI:** 10.1038/bjc.1990.264

**Published:** 1990-08

**Authors:** C. S. Heacock, R. M. Sutherland

**Affiliations:** Experimental Therapeutics Division of the Cancer Center, University of Rochester, School of Medicine and Dentistry, NY 14642.

## Abstract

**Images:**


Br. J. Cancer (1990), 62, 217-225 © Macmillan Press Ltd., 1990

### Enhanced synthesis of stress proteins caused by hypoxia and relation to altered cell growth and metabolism

C.S. Heacock* & R.M. Sutherland

*Experimental Therapeutics Division of the Cancer Center, and Departments of Radiation Oncology & Biophysics, University of Rochester, School of Medicine and Dentistry, Rochester, NY. 14642, USA.*

**Summary** Cultured cells maintained in very low oxygen levels alter their structure, metabolism and genetic expression. Culture conditions for cells were modified to minimise variation of nutrients and to allow normal survival levels after 24 h of hypoxic exposure. Under these hypoxic conditions, glucose consumption and lactate production rates were similar to aerobic rates until about 12 h after which the hypoxic rates increased. DNA and protein synthesis rates are continuously inhibited to about 48% or 55% of the respective aerobic rates. During this period of decreased protein synthesis, a set of proteins termed oxygen regulated proteins (ORPs), exhibits enhanced relative synthesis. The molecular weights of the five major ORPs are approximately 260, 150, 100, 80 and 33 kDa. While increased relative synthesis of oxygen regulated proteins is partly due to increased levels of mRNA which encode these proteins, the mechanism of enhanced synthesis of ORPs may be more complex.

Hypoxia and related pathophysiologic environmental and cellular metabolic changes can be determining factors for the effectiveness of treatment of tumours using radiation therapy (Hall, 1978), hyperthermia (Gerweck *et al.*, 1979) or chemotherapy (Teicher *et al.*, 1981). Cells are resistant to irradiation virtually immediately upon introduction of severe hypoxia and are sensitised quickly after oxygen addition (Michael *et al.*, 1973). Slower responses to hypoxia include increased hyperthermia and adriamycin resistance (Li & Shrieve, 1982; Smith *et al.*, 1980).

The most notable metabolic change during hypoxia is the increased rate of glucose consumption. Enhanced synthesis of selected proteins after hypoxia has been reported in plants (Guttman *et al.*, 1980) and mammalian cell lines (Sciandra *et al.*, 1984; Heacock & Sutherland, 1986). These stress proteins have been termed oxygen regulated proteins (ORPs) to denote proteins whose synthesis is enhanced during hypoxia and repressed after addition of oxygen. ORPs may be very similar to proteins synthesised after prolonged glucose deprivation (Sciandra *et al.*, 1984). To understand the molecular basis of resistance to various treatments and rationally plan therapeutic treatments, the basic biochemical events occurring during hypoxia must be studied. The degree of response to hypoxia may explain cell line variations of drug effects. For example, a DNA damaging agent may be expected to have differing hypoxic toxicities based on various hypoxic rates of DNA synthesis. Therefore, this report delineates the culture conditions and basic cellular responses, including the induction of ORPs, of cells observed during extreme hypoxia with carefully controlled glucose and hydrogen ion concentrations. Procedures and concepts presented here are being used for numerous other cell lines. A preliminary report of some of this work has appeared previously (Heacock & Sutherland, 1986).

### Methods

### *Cell culture*

EMT6/Ro mouse mammary tumour cells were maintained as previously described (Sutherland *et al.*, 1982) except that the growth medium contained the components for Basal Medium Eagle (BME) Hank's powdered medium plus the addition of 18 mM sodium bicarbonate, 10 mM HEPES, 15% fetal calf serum, $0.14\,g\,l^{-1}$ streptomycin sulphate and $10^5$ units $l^{-1}$ penicillin G with the pH adjusted to 7.3. HT-29 colon adenocarcinoma cells were cultured in Eagle's MEM media supplemented with 10% fetal calf serum and Chinese Hamster ovary cells were maintained in Ham's F-10 plus 10% fetal calf serum. Care was taken to omit potentially labile components (methionine, glutamine, glucose and serum) during preparation and storage, with their addition one day prior to initiation of an experiment. Cultures were routinely checked for the absence of mycoplasma (Chen, 1977). Additionally, rodent cell lines were free of human cell contamination by lactate dehydrogenase isozyme determination (Dietz & Lubrano, 1967).

Experimental cultures were initiated 20-24 hours before each experiment, except where noted, such that approximately $10^6$ cells existed per 100 mm diameter glass Petri dish. When dishes of differing size were used, the number of cells and volume of medium was changed proportionally to the measured surface area. Loosely attached and floating cells were removed by pipetting off the growth medium and BME rinse. Attached cells were removed by 5-10 min incubation at 37°C with 5 ml of 0.1% trypsin (Worthington). Trypsin was inactivated by addition of 5 ml of growth medium before determination of cell number and cell volume using a particle counter with channelizer (Coulter Electronics). Colony forming assays were performed as previously described (Sutherland *et al.*, 1982) with scored plates containing between 20 and 100 colonies with greater than 50 cells per colony.

Hypoxia, with the oxygen concentration less than 100 p.p.m., was achieved as previously described (Sutherland *et al.*, 1982) with 10 ml fresh growth medium placed on the cultures prior to the beginning of gas exchanges. The pH of the medium was determined immediately after opening of the gas chambers.

### *Biochemical methods*

*Glucose and lactic acid assays* Aliquots of media were centrifuged (1,000 *g*, 10 min) to remove intact cells and immediately frozen at −20°C for determination of glucose amount. Centrifuged media samples for lactic acid assays were diluted into perchloric acid, 3% final concentration, stored more than 30 min on ice then centrifuged in a microfuge (10,000 *g*, 5 min) and the clear supernatant stored at −20°C. Concentrations of glucose and lactic acid in media were stable for up to two months at −20°C; however, samples were not routinely assayed before two weeks due to interference by an unknown compound present in medium over hypoxic cultures (see Discussion). Glucose (Peterson &

*Present address: Cytogen Corporation, 201 College Road East, Princeton, NJ, USA.
Correspondence: R.M. Sutherland, SRI International, 333 Ravenswood Avenue, Menlo Park, California 94025, USA.
Received 16 August 1988; and in revised form 14 March 1990.

Young, 1968) and lactic acid (Rosenberg & Rush, 1966) assays which directly measured the reduction of $NADP^+$ or $NAD^+$ were utilised. Standards were purchased (Sigma Chemical Company, St Louis, MO) and a standard curve was completed for each set of determinations. A known quantity, 'spike', of glucose (or lactic acid) was added to aliquots from each sample for internal correction. All of the data presented have been adjusted with the correction factor less than 10% of the measured value.

*Protein, RNA and DNA synthesis* Total macromolecular synthesis was measured by both continuous incorporation and 30 minute pulse labelling of appropriate radioactive amino acid precursors. For protein synthesis measurements, $^{14}$C-leucine (350 mCi mmol$^{-1}$, Amersham) or $^{14}$C-amino acid mixture (Research Products International, approx. 50 mCi per milliatom of carbon) was added to complete growth medium at an initial concentration between 0.1–0.5 $\mu$Ci ml$^{-1}$ for both continuous and pulse labelling. After the indicated time period, the medium was removed, the culture rinsed twice with phosphate buffered saline (PBS), the dish placed on ice, and 0.3 M sodium hydroxide added for 10 min. The sodium hydroxide was pipetted off and another aliquot of 0.3 M sodium hydroxide used as a rinse. The combined sodium hydroxide solution was made to a final trichloroacetic acid (TCA) concentration of 10% and after 30 min on ice, the precipitate collected on glass fibre filters. The filters were washed with cold 10% TCA followed by ethanol, dried, immersed in 0.5% PPO (2,5 diphenyloxazole), 0.01% POPOP (1,4-*bis*-(2-(5-phenyloxazolyl))benzene) in toluene and the amount of radioactivity determined by scintillation counting with a counting efficiency of 90–95% using internal standards.

DNA synthesis was measured by continuous incorporation of $^{14}$C-thymidine (53 $\mu$Ci mmol$^{-1}$, Amersham) at 0.1–0.2 $\mu$Ci ml$^{-1}$ in complete growth media or pulse labelling with $^3$H-thymidine at 1 $\mu$Ci ml$^{-1}$. At the indicated time the radioactive medium was removed, the dish was placed on ice, rinsed twice with phosphate buffered saline, 5% cold TCA was added for 5 minutes (acid soluble fraction) then replaced with 0.3 M sodium hydroxide for 2 hours at room temperature (acid insoluble fraction). The amount of radioactivity of each fraction was determined by scintillation counting after addition of an aliquot to Scintiverse II (Fisher Scientific). Total RNA synthesis was determined by the same method as DNA synthesis except $^{14}$C-uridine (51 $\mu$Ci mmol$^{-1}$, Amersham) was added to a concentration of 0.2 $\mu$Ci ml$^{-1}$ in growth medium supplemented with 30 $\mu$M uridine to maintain a linear incorporation rate for continuous labelling experiments or 1 $\mu$Ci $^3$H-uridine ml$^{-1}$ supplemented growth medium for pulse labelling determinations.

*Other methods* ORP synthesis was routinely detected after the induction period (see Results) by replacing the medium with methionine deficient medium, plus 15% dialysed fetal calf serum and 10 $\mu$Ci ml$^{-1}$ $^{35}$S-methionine (1,000 Ci mmol$^{-1}$, Amersham) and incubating the culture for one hour in air. After labelling, the medium was removed, the cells washed twice with cold PBS and lysed in a small volume of 1% sodium dodecyl sulphate (SDS), 100 mM Tris pH 7.5, 25% glycerol, and 100 mM 2-mercaptoethanol. Solubilised samples were sheared through a pipette tip prior to determination of radioactive content following TCA precipitation, boiling and filtering (Panniers & Henshaw, 1984). Routine detection of ORPs was accomplished by one-dimensional SDS polyacrylamide gel electrophoresis (Lazarides & Granger, 1982) of samples containing equal radioactivity followed by autoradiography.

Free leucine concentration was determined by amino acid analysis after 3% sulphosalicylic acid treatment and centrifugation. Total protein per cell was determined using the Coomassie blue binding assay (Bradford, 1975) with bovine serum albumin as a standard.

Total RNA from hypoxic and aerobic cells was isolated by the guanidinium/caesium chloride method with mRNA purified by oligo(dT)cellulose chromatography (Maniatis *et al*., 1982). *In vitro* translation of the polyadenylated fraction was accomplished using a rabbit reticulocyte lysate *in vitro* translation kit (Bethesda Research Lab, Bethesda, MD or Promega Biotec, Madison, WI) with 50 $\mu$Ci $^{35}$S-methionine and 0.5–5 $\mu$g mRNA used per reaction. Non-linear incorporation rates occurred above 2 $\mu$g mRNA per reaction. After one hour incubation at 30°C, an equal volume of 2 × SDS lysis buffer was added and samples analysed on one-dimensional SDS gels (Lazarides & Granger, 1982). Rabbit globin mRNA (BRL) was used as a control. Total RNA and non-polyadenylated RNA were also translated *in vitro* using the above procedure except that the quantities used per reaction were 10–100 times the amounts described above.

### Results

### *Cell growth*

EMT6/Ro cell growth ceases upon initiating hypoxic conditions. After 24 hours of hypoxia total cell number is approximately the same as at the time of initiating hypoxia (Figure 1). Cell morphology appears normal for up to 12 hours of hypoxia. Between 1 and 2% of the total cell population is loosely attached or floating for periods of hypoxia up to 20 h. At 24 h, the percentage of floating cells rises to about 5%. Longer hypoxic periods result in decreasing total cell number with an increasing proportion of floating cells (e.g. 20% floating cells at 48 hours) and the remaining attached cells are extremely elongated. In spite of the tendency for hypoxic cells to round and float, those which remain attached are more difficult to remove from the surface by trypsinisation compared to aerobic controls.

Aerobic growth rates obtained with the amount of medium used for these studies (0.13 ml cm$^{-2}$) are identical to aerobic growth rates using larger medium volumes (0.22 ml cm$^{-2}$) which is used for maintenance of stock cultures and colony forming assays. With smaller amounts of medium (0.09 ml cm$^{-2}$) the initial growth rate remains the same but a subconfluent plateau density is reached at about 50 hours.

The gas exchange procedure used to achieve extreme hypoxia is performed at room temperature. Therefore time is required, after placing the chambers at 37°C at 0 h, for the cultures to reach a temperature compatible with cell growth. In paired time point experiments, no difference in growth rate after 4 h was noted between aerobic cultures subjected to the gas exchanges and cultures continuously at 37°C (data not shown).

**Figure 1** EMT6 attached cell growth under aerobic (solid) or hypoxic (open) culture conditions. Cell number is relative to cultures prior to 2.5 h of gas exchanges and placement in 37°C room at 0 h. Data points are the mean of 3–5 triplicate determinations with bars indicating standard deviation. Lines are least squares fit of the means giving a 15 h doubling time for the aerobic cultures. Initial cell number ranged between 2–4.2 × $10^5$ cells per 49 mm glass Petri dish with 2.9 ml growth medium per Petri dish.

*Survival*

Clonogenic plating efficiency of euoxic and hypoxic cells has been very constant over the three year period of data collection, provided the cell line has been maintained in exponential phase for less than 25 passages and the medium has increased buffering capacity to guard against a pH drop. The plating efficiency (PE) of the attached hypoxic cultures is similar but slightly lower than the aerobic attached cells (Figure 2). With up to 24 h of hypoxia greater than 50% of the attached cells are reproductively competent. The PE of the aerobic floating and loosely attached cells is reasonably constant at approximately 30%, but the PE of the hypoxic counterparts decreases steadily with increasing time in hypoxia. However, the PE of the total population (attached plus floaters) is almost the same as the attached due to the small percentage of floating cells. At times less than 24 h the attached population is greater than 98% of the total population.

*Cell density*

Many biochemical procedures (e.g. isolation of RNA or specific proteins) require a large number of cells; therefore the greater the cell density the more efficient cell acquisition becomes. The constraint imposed here is that the cells must remain reproductively viable after hypoxic treatment.

When increased numbers of cells are plated one day prior to hypoxic treatment, the growth curve (Figure 3a) and plating efficiency (Figure 3b) of cells maintained at densities between 10 and 30% of the confluency value of $3 \times 10^6$ cells per 49 mm are nearly normal through 24 hours. However, higher densities of confluency result in decreased survival. These decreased survival values occur when the pH of the culture medium was 7.1–7.2 and amounts of glucose exist which are sufficient for aerobic cultures to survive for several days (unpublished data, C Heacock). Due to the unknown mechanism of hypoxic cell death in high density cultures, all of the characteristics presented in this paper are derived from cell densities between 5–20% of the confluency value.

**Figure 2** Clonogenic plating efficiency of EMT6 cells under aerobic (solid) or hypoxic (open) conditions. Circles denote attached cells and triangles signify floating plus loosely attached cells. Circles are the mean of 3–5 experiments with bars showing standard deviation. Triangles are the average of two experiments.

*pH, total protein/cell, glucose and lactic acid*

The pH of the growth medium remains constant for up to 48 h within each experiment in either hypoxic or euoxic conditions, however, among experiments the absolute value

**Figure 3** **a**, Cell growth density effect on attached EMT6 cell growth under aerobic (solid) or hypoxic (open) conditions. Symbols denote various initial attached cell numbers per 49 mm glass Petri dish. Each dish contained 2.9 ml growth medium. Confluency is reached at approximately $3 \times 10^6$ cells although growth continues beyond this density. Data points are the average of triplicate determinations. **b**, Cell density effect on subsequent clonogenic plating efficiency. Symbols same as **a**. Data not corrected for cell loss before plating.

of the pH varies ± 0.1 pH unit depending on the $CO_2$ content of the compressed gas.

Total protein per cell does not increase significantly during hypoxia ($218 \pm 43 \times 10^{-12}$ g protein per cell in air and $230 \pm 56 \times 10^{-12}$ g protein per cell after 12 h of hypoxia at matched cell densities at the time of cell harvest for protein content determination). Similar results were also found in two other rodent (CHO, MSV-3T3) and five human cell lines (A431, HT29, CaSKi, Coll2, JAR). Therefore, data are expressed on a per cell basis because cell number is determined at several points during each experiment presented here.

The amounts of glucose and lactic acid (Figure 4) in the medium are virtually identical between euoxic and hypoxic cultures with approximately half of the glucose remaining after 48 hours under these low cell density conditions. However, since the number of hypoxic cells does not change, unlike the aerobic conditions where growth continues, the rates of glucose utilisation and lactate production on a per cell basis are quite different (Table I). The glucose consumption rate of hypoxic cultures for the initial 12 h is similar to the glucose consumption rate for aerobic cultures. With further hypoxic exposure, the glucose consumption rate appears to increase compared to the stable aerobic rate. Lactate production rates for hypoxic cultures follow a similar pattern, initially similar to the aerobic cultures with a subsequent rate increase. Generally similar results for glucose consumption and lactate production have been observed for CHO cells.

**Figure 4** Glucose (circles) and lactic acid (squares) concentration in growth medium over aerobic (solid) and hypoxic (open) cultures. Data scaled to initial cell number of $3 \times 10^5$ cells per 49 mm glass Petri dish, 2.9 ml medium per dish, unused medium equal to 6.55 mM glucose and 1.69 mM lactic acid. Actual cell density varied between 2 and $4.2 \times 10^5$ cells per 49 mm Petri dish. Points are mean of 3–7 determinations with bars equal to standard deviations.

**Table I** EMT6 cell glucose consumption and lactic acid production

| | *Rate of Glucose consumption* ($10^{-13}$ *mol cell*$^{-1}$ *h*$^{-1}$) | *Rate of lactic acid production* ($10^{-13}$ *mol cell*$^{-1}$ *h*$^{-1}$) |
|---|---|---|
| Aerobic (0–24 h) | 4.7±4.5 | 9.7±2.7 |
| Hypoxic (0–12 h) | 4.1±6.3 | 11.4±7.0 |
| (12–24 h) | 9.1±3.3 | 29.8±5.8 |

Rates are means ±s.d. of the rate of change of metabolite concentration/cell over 4 h intervals from individual experiments. Aerobic rates are calculated using a formula which adjusts for exponential growth (Shrieve *et al.*, 1983).

*Protein synthesis*

Hypoxic environments lead to a reduction of total protein synthesis even when corrected for the lack of cell growth (Figure 5). The rate of total protein synthesis per hypoxic cell is 55 ± 8% ($n = 5$) of the rate per aerobic cell. These results are also seen using $^{14}$C-labelled amino acid mixtures (data not shown).

This reduction in protein synthesis rate is not due to an artefact from a decrease in the leucine pool size which could happen if leucine were used as an energy source. However, varying the concentration of leucine in the medium shows that above 0.05 mM the medium is saturating for leucine (i.e. after correcting for specific activity, a constant amount of radioactivity is incorporated per cell for increasing amounts of leucine in the growth medium, data not shown). All experiments described here were performed in BME containing 0.2 mM leucine (26 mg $l^{-1}$) plus serum giving a total free leucine concentration of about 0.25 mM. Determination of free amino acid concentrations did not show any large differences in leucine concentrations (or other amino acids) between aerobic medium, hypoxic medium or medium without cells incubated for 12 hours (data not shown).

The results reported here are for continuously labelled cultures, i.e. radioactive leucine added prior to initiation of gas exchanges. The rate of protein synthesis, between 12 and 14 h of hypoxia, determined using a pulse label administered at 12 h of hypoxia and harvested at 7 time points during the following 2 hours of hypoxia, was very similar to the rate determined by the continuous label procedure over the same time period (data not shown). Additionally, 30 minute pulse labelling results taken at several time points between 0 and 24 h of hypoxia are very similar to the data presented (data not shown). Similar results were obtained for CHO cells.

**Figure 5** EMT6 cell protein synthesis under aerobic (solid) and hypoxic (open) atmosphere. Symbols are the mean of triplicate determinations from one representative experiment with cumulative rate data. $^{14}$C-leucine added to 0.25 μCi $ml^{-1}$ at −2.5 h. Initial cell number equalled = $3 \times 10^5$ per 49 mm glass Petri dish. Counting efficiency = 93%.

*DNA and RNA synthesis*

Rates of incorporation of DNA and RNA precursors per dish are greatly decreased during hypoxia. When corrected for the lack of cell growth, uridine incorporation (Figure 6) shows only a slight decrease in rate (88 ± 14%, $n = 3$) when compared to aerobic cultures, but a reproducible 2 hour lag is noted. Precursor incorporation into DNA (Figure 7) is inhibited (47 ± 4%, $n = 3$) compared to aerobic cultures. As with protein synthesis, the continuous label data presented is very similar to 30 minute pulse label data.

*Oxygen-regulated protein induction*

Enhanced synthesis of several selected proteins is noted after initiation of extreme hypoxia (Figure 8). The molecular weights of the major oxygen regulated proteins are 260, 150, 100, 80, and 33 kDa. ORP 150 is very difficult to visualise in EMT6 cells but in other cell lines is one of the major cellular protein bands (Heacock, unpublished data). Other bands, at 20–24, 35–37, 43–45 and 60 kDa, occasionally are detected as ORPs but are not consistent among experiments or various cell lines (data not shown). The most dramatic decreased level of synthesis is noted in the band that corresponds to one of the major proteins induced by heat shock (70 kDa). Detection of ORPs does not depend greatly on the selection of radioactive amino acid except for a slight enhancement of ORP 80 using a $^{14}$C-amino acid mixture implying a lower than average methionine content. No additional low molecular weight ORPs (10–40 kDa) are detected using a $^{14}$C-amino acid labelling mixture and high percentage polyacrylamide gels. Labelling immediately after the hypoxia period gives very similar results to labelling during the hypoxic period except for a slight diminution of ORP 260.

The kinetics of ORP synthesis are different for the various proteins (Figure 9) with the synthesis of the 260 kD protein being enhanced very quickly and is detectable between 0 and 2 h. The other ORPs are detected after 3–4 h of hypoxia. In general, the synthesis rates of the ORPS continuously increases until 8–10 h. However, with hypoxia exposures greater than 12 h, the level of enhancement at a particular

**Figure 6** EMT6 cell incorporation of $^{14}$C-uridine in aerobic (solid) and hypoxic (open) cultures. Symbols and conditions are identical to Figure 5 except $^{14}$C-uridine was added to a final concentration of 0.17 μCi ml$^{-1}$ plus medium supplement with 30 μM non-radioactive uridine to maintain a constant rate of incorporation.

**Figure 7** EMT6 cell incorporation of $^{14}$C-thymidine in aerobic (solid) and hypoxic (open) cultures. Symbols and conditions are identical to Figure 6 except $^{14}$C-thymidine was added to 0.2 μCi ml$^{-1}$.

time point varies between experiments and between time points even though the ORPs are always detectable at greater than aerobic synthesis rates and HSP70 is lesss than the aerobic level. Using existing methodologies it is only possible qualitatively to state that after 12 h of hypoxia the synthesis rate for ORP 80 decreases while the rate for ORP 33 continuously increases, and the rate for ORP 260 is reasonably constant. Other cell lines have slightly different absolute kinetics; however, the kinetics of the ORP synthesis rates relative to each other follow the same pattern which is ORP 260 first, ORP 33 last, and the others intermediate.

Induction of specific mRNA can be shown directly by isolating total cellular RNA and demonstrating enhanced relative *in vitro* synthesis of that protein. *In vitro* translation of total RNA or the polyadenylated RNA fraction from hypoxia cells compared to that from aerobic cells (Figure 10) clearly shows enhanced synthesis of all five ORPs and reduced synthesis of the 70 kDa heat shock protein. ORPs are not detected from *in vitro* translations of non-polyadenylated RNA fractions.

### Discussion

The ultimate goal in devising a system in which to study the effects of hypoxia is to create an environment in which the only variable is the oxygen concentration. The most difficult variable to control was pH. Anaerobic conditions usually induce cells to consume glucose at higher rates (Pasteur effect) which leads to higher lactic acid production which lowers the pH. Additional buffering capacity in the medium plus a low cell density resulted in both a relatively stable pH and glucose concentration throughout the period tested. Many nutrients are necessary for balanced cellular metabolism, but glucose consumption increases dramatically

**Figure 8** Oxygen regulated protein detection during and after hypoxia. Lanes 1 and 2 cultures labelled with $^{14}$C-amino acid mixture. Lanes 1 and 3 are from aerobic cultures. The samples for lanes 2, 4, and 5 are from cultures which were hypoxic for 12 hours. The samples for lanes 2 and 4 were labelled during hypoxia (hours 8–12) but the sample for lane 5 was labelled for one hour in air following hypoxia for 12 h. Lanes 3, 4 and 5 are from cultures labelled with $^{35}$S-methionine (50, 100, and 10 $\mu$Ci ml$^{-1}$ respectively). Radioisotope concentrations and labelling times were adjusted to equalise roughly the total amount of radioactivity incorporated per cell. Equal amounts of radioactivity ($10^6$ c.p.m.) were loaded in each lane with exposure for 2 days prior to development. Left margin denotes the positions of molecular weight markers (Bio-Rad). Arrows indicate ORPS.

**Figure 10** Autoradiograms of *in vitro* translation of purified HT-29 mRNA. The two right lanes depict the $^{35}$S-methionine patern of proteins synthesised in intact cells after 12 h of hypoxia. The two left lanes show *in vitro* translation of total polyadenylated RNA 0.5 $\mu$g per assay. Approximately equal amounts (68,000 c.p.m.) of TCA precipitable radioactivity were loaded in each lane. Exposure time was 7 weeks. all five ORPs are indicated by lines. For presentation clarity, results from HT-29 cells are shown instead of those from EMT6 cells which do not reproducibly show the higher molecular weight ORPs.

**Figure 9** Kinetics of EMT6 cell ORP induction. Autoradiogram after labelling with $^{35}$S-methionine (10 $\mu$Ci ml$^{-1}$) for 1 h after hypoxic treatment period shown in hours on top margin. Approximate location of ORPs are indicated on side margins. HSP refers to 70 kDa heat shock protein.

under hypoxic conditions. Therefore, if glucose levels are relatively unchanged, it is assumed that other unknown critical nutrients also are not responsible for the effects during early stages of hypoxia, thus the results noted here are presumably due to oxygen deprivation either directly or indirectly.

The extreme hypoxic conditions used in this study resulted in rapid cessation of EMT6/Ro cell growth. The cell cycle distribution of these cells essentially remains the same as exponentially growing aerobic cultures of EMT6/Ro cells (unpublished observations) as well as other cell lines (Shrieve *et al.*, 1983). However, continued progression of cells initially in mitosis (originally 5–10% of the total population) with a block in all other phases giving rise to a slight increase in cell number cannot be ruled out due to the small percentage of the population being affected (Born *et al.*, 1976; Rice *et al.*, 1985; Petterson *et al.*, 1986). The gradual change in EMT6/Ro morphology (from a flat, triangular shape to stretched spindle shape to finally rounding before detachment) is similar to other cell lines (Jacobson, 1981; Heacock, unpublished results). Jacobson noted that an initial cell volume decrease was followed by a slow cell swelling for hypoxic cells (Jacobson, 1981). During this period, hypoxic cells are characterised by swollen mitochondria, dilated endoplasmic reticulum and pronounced lipid vesicles (Jacobson *et al.*, 1985). These morphological observations are consistent with early potassium efflux resulting from calcium release from hypoxic mitochondria (Chien *et al.*, 1978). Subsequent swelling may reflect the influx of sodium and water since $Na^+/K^+$ ATPase activity is diminished (Johnstone *et al.*, 1985). Throughout these morphological changes, total protein per cell is fairly constant for EMT6 cells as well as other cell lines (Petterson *et al.*, 1986; Jacobson, 1981; Heacock, unpublished results).

Plating efficiency remains at about the same level as the aerobic controls for about the first 24 hours of hypoxia with a slow decline afterwards which is similar or slightly higher compared to other reports for cells incubated at 37°C (Shrieve *et al.*, 1983; Born *et al.*, 1976). The glucose concentration in the medium does decrease slightly over the period studied but aerobic EMT6/Ro cells survive several days of virtually no glucose with no change in viability (Heacock, unpublished observation). At higher cell densities hypoxic cells die faster implying either a depletion of a critical substance other than glucose (supported by data from hypoxic cells deprived of glucose to be presented elsewhere), or accumulation of toxic products.

The absolute values of aerobic glucose consumption rates reported here are similar to other determinations in EMT6/Ro cells (Ling, 1986; Freyer & Sutherland, 1985) as well as other cell lines (Shrieve *et al.*, 1983). Under hypoxic conditions, glucose consumption rates normally increase. However, glucose consumption per cell does not appear to increase immediately upon initiation of extreme hypoxia as the levels of glucose in the growth medium are virtually constant, but requires several hours before cells switch from aerobic glycolysis to anaerobic glycolysis. Figure 5a shows glucose concentration versus time but since cell number does not change during hypoxia the slope change after 12 hours indicates the increase in glucose consumption rate. However, the small changes in glucose concentration in low cell density result in large errors when rates were calculated.

The classical method of glucose consumption (and lactic acid production) rate measurements involves high density suspension cultures for a short period of time sufficient to achieve an easily measured change in glucose concentration. As we have shown during hypoxia, high density cultures have a different growth and survival response than low density cultures. The compromise we made was to choose a precise biological system with less precise results. However, the results obtained are comparable to those done by classical procedures performed in the same lab, with the same cells for the initial time points. Our attempt was to extend these measurements to study long term hypoxic conditions. The conclusion from our data is that after a considerable length of time, cells are continuing to adapt metabolically to hypoxia as evidenced by the slow change in glucose consumption and lactate production. If indeed adaptation requires a considerable period of time, then ATP production would be expected to decrease and total energy levels drop during adaptation. After full adaptation, energy levels would be expected to reach a new steady state level probably with a decrease in both energy production and energy consumption due to lack of cell growth and decreased macromolecular synthesis.

It was noted that medium from hypoxic cultures of several cell lines contained a compound which chemically reduced the tetrazolium dye which was used as the final coupled reaction in a colorimetric glucose assay (Carroll *et al.*, 1970). The amount of dye reduction was not related to the amount of glucose present thereby giving erroneously high measurements. This chemical reduction was cell line dependent and slowly disappeared, possibly oxidised, at −20°C. After two weeks of storage, the interference was minimal. Therefore, the data presented here are derived from enzymatic assays which measure direct NADP reduction for glucose determinations or $NAD^+$ reduction for lactic acid determination. Both of these assays measured the correct values as determined by enzyme and substrate concentration titrations and addition of known amounts of standards to the samples.

*Protein synthesis*

Extreme hypoxia has been shown to inhibit total protein synthesis about 87% in V79 cells (Jacobson, 1981), 64% in NHIK 3025 cells (Petterson *et al.*, 1986), 40% in EMT6/SF cells (Shrieve *et al.*, 1983), 45% in EMT6/Ro cells (this report) and in other rodent and human cell lines which we have studied (data not shown). While no reasons for hypoxic inhibition of protein synthesis have been previously proposed, we have ruled out several possibilities. The extent of inhibition is constant after 2 h. Therefore, whatever causes the effect must be completed or have reached a steady state level before 2 h. The inhibition between 0 and 2 h seen in both aerobic and hypoxic cultures is presumed to be temperature mediated because of the gassing technique required to obtain hypoxia. Protein synthesis is known to be inhibited by the lack of a single amino acid (Pain *et al.*, 1980). However, fresh medium was placed over the cultures immediately prior to initiation of hypoxia. Therefore, the concentration of amino acids is presumed to be sufficient at early stages of hypoxia. This is true at least for the first 12 h of hypoxia as verified by analysis of free amino acids. The increased metabolism of leucine, not leading to incorporation into protein, is not a likely explanation because both the hypoxic and aerobic rates saturate at leucine concentrations greatly below those normally used for growth medium even though the absolute rates differ at saturating leucine concentration. Further, the choice of precursor (leucine or a commerical mixture of amino acids) or pulse labelling with leucine gives similar results. An increased level of phosphorylation of eurkaryotic initiation factor-2 (eIF-2) or inactivations of eIF-4F has been postulated for the inhibition of protein synthesis during hyperthermia (Panniers *et al.*, 1985). The possibility that modulation of eIF-2 and eIF-4F activity also contributes to hypoxic protein synthesis inhibition has not been investigated.

ATP levels decrease rapidly after initiation of hypoxia (Gillies *et al.*, 1982) which would lead to inhibition of protein synthesis. Also, hypoxic cell protein synthesis can be completely inhibited at normal ATP levels, presumably due to accumulation of the inhibitory products of ATP hydrolysis (Freudenberg & Majar, 1971). ADP and AMP appear to accumulate during hypoxia (Gillies *et al.*, 1982). Therefore protein synthesis regulation by changes in the adenine nucleotide pools is possible; however, others (Probst *et al.*, 1988) have reported no change in adenylate energy charge during hypoxia.

Thymidine incorporation into DNA after 8 hours of hypoxia has been reported to be about 31% (Jacobson *et al.*, 1985) and 15% (Shrieve *et al.*, 1983) of the aerobic amount. However, Shrieve found a continuously decreasing rate of incorporation with increasing time of hypoxia. Our rate of 48% in EMT6/Ro is somewhat closer to the aerobic rate but also remains constant up to 24 hours under our conditions. The intracellular thymidine pool size is reasonably constant because the amount of radioactivity in the acid soluble fraction was similar throughout the hypoxic period, and therefore precursor availability is presumed not to be a limiting factor. Recently it was shown (Probst *et al.*, 1988) that the essential cause of the decrease in DNA synthesis associated with hypoxia was the suppression of initiation while DNA chain elongation remains unaffected.

Incorporation rates of uridine are similar for aerobic and hypoxic cells. The lag period during hypoxia may be interpreted as either a temporary absence of a necessary factor (such as decreased triphosphate pool size) or as a temporary presence of an inhibitor (e.g. inappropriate ionic conditions). However, a uridine supplement was required to maintain a constant radioisotope incorporation rate under both conditions, and may indicate that EMT6/Ro cells utilise uridine for purposes other than RNA synthesis. Isolating RNA after labelling should clarify this possibility.

The continued, relatively high, synthetic rates of DNA, RNA and protein under hypoxic conditions are somewhat surprising during a lack of cell growth (no increase in cell number or total protein per cell). Recently, it was shown (Petterson *et al.*, 1986) that at 3 hours of hypoxia protein degradation increases slightly and protein synthesis decreases greatly resulting in a constant amount of protein per cell. Similar turnover rates have not been determined for RNA or DNA but DNA damage could be accumulating during hypoxia. Therefore, presumably a residual amount of DNA synthesis is required for continued cell survival in hypoxia. The wide variations among cell lines in the inhibition of the amount of DNA synthesis during hypoxia, and probably their ability to resume normal synthesis during reoxygenation (Rice *et al.*, 1986), may explain the cell line dependent variations in sensitivities to DNA directed drugs.

Two technical difficulties were present in attempting to detect hypoxia induced proteins: (1) protein synthesis is inhibited during hypoxia and (2) high specific activity amino acids must be used for efficient detection of specific protein synthesis. The solution was to replace medium with methionine-free medium plus high specific activity $^{35}$S-methionine and incubate for one hour in the presence of oxygen. The same five major ORPs are detected using this methodology compared to labelling cells with large amounts of radioactivity during hypoxia. The relative rates of synthesis of these proteins are similar except for ORP 260 whose synthesis diminishes rapidly after aeration (unpublished observation). Therefore, this protocol may be used for quantitating the kinetics of ORP synthesis but will slightly underestimate the rate ORP 260 synthesis.

Our operational definition of oxygen regulated proteins depends on the detection of increased synthesis of a particular band (or spot on a 2-D gel) relative to other bands. The enhanced synthesis of ORPs relative to other proteins may arise through three general regulation processes: (1) increased transcription of the ORP genes, (2) altered RNA processing and stability, or (3) ribosome binding and translation into ORPs. *In vitro* translation of hypoxic polyadenylated RNA from mouse EMT6 cells consistently shows more ORP 33 and 80 compared to aerobic polyadenylated RNA but higher molecular weight proteins (both ORP and non-ORP) have been difficult to detect in both hypoxic and aerobic EMT6 samples. In human HT-29 cells, all five ORPs can be identified from *in vitro* translation of hypoxic polyadenylated RNA and total RNA.

Inhibiting RNA synthesis in cells with actinomycin D before and during hypoxia results in a relative decrease in only ORP 80 synthesis in several cell lines. These studies suggest that regulation of both transcription and translation may play an important role in the relative enhanced synthesis of ORPs.

Removal of oxygen has been previously shown to lead to enhanced synthesis of two proteins in CHO cells (Sciandra *et al.*, 1984). Proteins which co-migrate, at molecular weights of 80 and 100, with the CHO proteins are also induced in EMT6/Ro cells and several other cell lines (data not shown). The induction kinetics of the 80 and 100 kD proteins described here are slightly more rapid than those described by Sciandra *et al.* (1984), possibly due to a more rapid removal of oxygen. The two previously identified proteins were shown to have very similar polypeptide chains to the two major proteins induced by glucose deprivation (Sciandra *et al.*, 1984). However, the glucose regulated proteins are underglycosylated and migrate at a correspondingly lower molecular weight (Pouyssegur & Yamada, 1978). Further characterisation of these proteins has been accomplished by Welch *et al.* (1983), Munro & Pelham (1986), Subjeck & Shyy (1986), Mazzarella & Green (1987), Lee (1987) and Hendershot *et al.* (1988).

Three additional ORPs of molecular weights 260, 150, and 33 kDa are detected in EMT6/Ro cells as well as other rodent and human cell lines (Heacock & Sutherland, 1986). The qualitative kinetics of continual relative increase in synthesis rates during hypoxia of the four lower molecular weight ORPs are very similar suggesting a similar mechanism of enhanced synthesis. The behaviour of ORP 260 appears distinct from the other ORPs. The enhanced synthesis of ORP 260 is detected very quickly, the synthesis rate increases more rapidly, and the synthesis rate appears to plateau. Also, the addition of actinomycin D prior to and continued exposure through hypoxia results in greatly enhanced synthesis of ORP 260. A further distinguishing characteristic of ORP 260 is its apparent decrease after heating or prolonged storage at −20°C. Possibly this instability indicates a susceptibility to oxidation which leads to an aggregate which does not enter the gel since no large increase in any faster migrating band is noted. The possiblity that this protein may serve as a precursor to the other ORPs has not been ruled out but the continued accumulation of ORP 260 during hypoxic periods greater than 10 hours, as visualised by Coomassie blue staining, during a period of reasonably constant synthesis, makes this hypothesis unlikely.

Based on Coomassie blue stained gels, ORPs 80 and 100 are relatively abundant proteins in aerobic cultures. Thus additional protein accumulation is difficult to detect. ORP 260 and 150 are very barely visible in aerobic cultures but in cell lines where significant enhanced synthesis is observed, accumulation is apparent. The amount of apparent accumulation of ORP 33 is intermediate in both aerobic and hypoxic cultures.

The functions of these hypoxia-induced stress proteins are unknown. Some, at least ORPs 80 and 100, which are similar to the glucose regulated proteins, may be involved in protein processing and transport (Munro & Pelham, 1986; Mazzarella & Green, 1987; Lee, 1987; Hendershot *et al.*, 1988). Drug resistance induced by hypoxia or glucose starvation correlates with the presence of some of these stress proteins (Shen *et al.*, 1987; Wilson *et al.*, 1989; Wilson & Sutherland, 1989). Antibodies against the major ORPs are being prepared to localise these proteins. One possibility is localisation of some ORPs on the nucleolus to protect RNA production similar to heat shock proteins 70 and 110 (Welch & Feramisco, 1985; Subjeck *et al.*, 1983).

The existence of additional ORPs is probable, but the five proteins reported here are detected reproducibly in many cell lines. Bands at 20–24, 35–37, 43–45 and 60 kDa have been noted but are not reproducible and have not been observed in all cell lines tested. These lower molecular weight bands may be partial degradation products.

A mechanism which encompasses most of the observations presented here relies on a hypoxia induced ionic imbalance proposed by Hochachka (1986). Lowered ATP production occurs during adaptation to anaerobic glycolysis which leads to sodium–potassium imbalance which could rapidly inhibit DNA and protein synthesis. Later effects within the first few hours involve calcium fluxes leading to the changes in mitrochondria structure and general cell swelling and enhanced synthesis of ORPs.

The authors would like to thank Dr Fred Pierce for amino acid analyses, Martin Siegrist for densitometry of gels, and Chris Edler for assisting with the macromolecular synthesis experiments. We gratefully acknowledge the helpful discussions with Dr Edgar Henshaw and with members of the Experimental Therapeutics Division, especially Drs James Sciandra and Robert Wilson. We also extend our appreciation to Mary LeRoy-Jacobs and others for manuscript typing and to Pat Grant for editorial assistance. This research was supported by NIH Grant T32-CA09363 and American Cancer Society Grant PDT-320.

### References

BORN, R., HUG, O. & TROTT, K.-R. (1976). The effect of prolonged hypoxia on growth and viability of Chinese hamster cells. *Int. J. Radiat. Oncol. Biol. Phys.*, **1**, 687.

BRADFORD, M. (1976). A rapid and sensitive method for the quantitation of microgram quantities of protein utilizing the principle of protein-dye binding. *Anal. Biochem.*, **72**, 248.

CARROLL, J.J., SMITH, N. & BABSON, A.L. (1970). A colorimetric serum glucose determination using hexokinase and glucose-6-phosphate dehydrogenase. *Biochem. Med.*, **4**, 171.

CHEN, T.R. (1977). In situ detection of mycoplasma contamination in cell culture by fluorescent Hoechst 33258 stain. *Exp. Cell. Res.*, **104**, 255.

CHIEN, K.R., ABRAMS, J., SERRONI, A., MARTIN, J.T. & FARBER, J.L. (1978). Accelerated phospholipid degradation and associated membrane dysfunction in irreversible, ischemic liver cell injury. *J. Biol. Chem.*, **253**, 4809.

DIETZ, A.A. & LUBRANO, T. (1967). Separation and quantitation of lactic dehydrogenase isoenzymes by disc electrophoresis. *Anal. Biochem.*, **20**, 246.

FREUDENBERG, H. & MAGER, J. (1971). Studies on the mechanism of protein synthesis induced by intracellular ATP depletion. *Biochim. Biophys. Acta*, **232**, 537.

FREYER, J.P. & SUTHERLAND, R.M. (1985). A reduction in the in situ rates of oxygen and glucose consumption of cells in EMT6/Ro spheroids during growth. *J. Cell. Phys.*, **124**, 516.

GERWECK, L.E., NYGAARD, T.G. & BURLETT, M. (1979). Response of cells to hyperthermia under acute and chronic hypoxic conditions. *Cancer Res.*, **39**, 966.

GILLIES, R.J., OGINO, T., SHULMAN, R.G. & WARD, D.C. (1982). $^{31}$Nuclear magnetic resonance evidence for the regulation of intracellular pH by Ehrlich ascites tumor cells. *J. Cell Biol.*, **95**, 24.

GUTTMAN, S.D., GLOVER, C.V.C., ALLIS, C.D. & GOROVSKY, M.A. (1980). Heat shock, deciliation and release from anoxia induce and synthesis of the same set of polypeptides in starved T. pyriformis. *Cell*, **22**, 299.

HALL, E.J. (1978). *Radiobiology for the Radiologist*, 2nd edition. Harper & Row: New York.

HEACOCK, C.S. & SUTHERLAND, R.M. (1986). Induction characteristics of oxygen regulated proteins. *Int. J. Radiat. Oncol. Biol. Phys.*, **12**, 1287.

HENDERSHOT, L.M., TING, J. & LEE, A.S. (1988). Identity of the immunoglobulin heavy-chain-binding protein and the role of post translation modifications in its binding function. *Mol. Cell Biol.*, **8**, 4250.

HOCKACHKA, P.W. (1986). Defense strategies against hypoxia and hypothermia. *Science*, **231**, 234.

JACOBSON, E.A. (1981). PhD Thesis, University of Western Ontario, London, Canada.

JACOBSON, E.A., HUTCHINSON, K., INCH, W.R. & TUSTINOFF, E.R. (1985). Ultrastructural changes in V79 hamster lung fibroblasts during hypoxic exposure. *Virchows Arch.*, **49**, 23.

JOHNSTONE, S.A., SCHURCH, S., MCIVER, D.J.L., JACOBSON, E.A. & TUSTINOFF, E.R. (1985). Membrane glycoprotein and surface free energy changes in hypoxic fibroblast cells. *Biochim. Biophys. Acta*, **815**, 159.

LAZARIDES, E. & GRANGER, B.L. (1982). Preparation and assay of the intermediate filament proteins desmin and vimentin. *Methods Enzymol.*, **85**, 488.

LEE, A.S. (1987). Coordinated regulation of a set of genes by glucose and calcium ionophore in mammalian cells. *Trends Biochem. Sci.*, **12**, 20.

LI, G.C. & SHRIEVE, D.C. (1982). Thermal tolerance and specific protein synthesis in Chinese hamster fibroblasts exposed to prolonged hypoxia. *Exp. Cell Res.*, **142**, 464.

LING, L. (1986). PhD Thesis, University of Rochester, Rochester, NY, USA.

MANIATIS, T., FRITSCH, E.F. & SAMBROOK, J. (1982). *Molecular Cloning: a Laboratory Manual.* Cold Spring Harbor Laboratory: Cold Spring Harbor, NY.

MAZZARELLA, R.A. & GREEN, M. (1987). ERp99, an abundant, conserved glycoprotein of the endoplasmic reticulum, is homologous to the 90-kDa heat shock protein (hsp 90) and the 94-kDa glucose regulated protein (GRP 94). *J. Biol. Chem.*, **262**, 8875.

MICHAEL, B.D., ADAMS, G.E., HEWITT, H.B., JONES, W.B.G. & WATTS, M.E. (1973). A posteffect of oxygen in irradiated bacteria: a submillisecond fast mixing study. *Radiat. Res.*, **54**, 239.

MUNRO, S. & PELHAM, H.R.B. (1986). An Hsp 70-like protein in the ER: identity with the 78 kDa glucose-regulated protein and immunoglobulin heavy chain binding protein. *Cell*, **46**, 291.

PAIN, V.M., LEWIS, J.A., HUVOS, P., HENSHAW, E.C. & CLEMENS, J.M. (1980). The effects of amino acid starvation on regulation of polypeptide chain initiation in Ehrlich ascites tumor cells. *J. Biol. Chem.*, **225**, 1486.

PANNIERS, R. & HENSHAW, E.C. (1984). Mechanism of inhibition of polypeptide chain initiation in heat-shocked Ehrlich ascites tumour cells. *Eur. J. Biochem.*, **140**, 209.

PANNIERS, R., STEWART, E.B., MERRICK, W.C. & HENSHAW, E.C. (1985). Mechanism of inhibition of polypeptide chain initiation in heat-shocked Ehrlich cells involves reduction of eukaryotic initiation factor 4F activity. *J. Biol. Chem.*, **260**, 9648.

PETERSON, J.I. & YOUNG, D.S. (1968). Evaluation of the hexokinase/glucose-6-phosphate dehydrogenase method of determination of glucose in urine. *Anal. Biochem.*, **23**, 301.

PETTERSEN, E.O., JUUL, N.O. & RONNING, O.W. (1986). Regulation of protein metabolism of human cells during and after acute hypoxia. *Cancer Res.*, **46**, 4346.

POUYSSEGUR, J. & YAMADA, K.M. (1978). Isolation and immunological characterization of a glucose-regulated fibroblast cell surface glycoprotein and its nonglycosylated precursor. *Cell*, **13**, 139.

PROBST, H., FCHISSER, H., GEKELER, V. & KIBNZLE-PFEILSTRICKER, H. (1988). Oxygen dependent regulation of DNA synthesis and growth of Ehrlich ascites cells. *Cancer Res.*, **48**, 2053.

RICE, G.C., HOY, C. & SCHIMKE, R.T. (1986). Transient hypoxia enhances the frequency of dihydrofolate reductase gene amplification in Chinese hamster ovary cells. *Proc. Natl Acad. Sci. USA*, **83**, 5978.

RICE, G.C., SPIRO, I.J. & LING, C.C. (1985). Detection of S-phase overreplication following chronic hypoxia using a monoclonal anti-BrdVrd. *Int. J. Radiat. Oncol. Biol. Phys.*, **11**, 1817.

ROSENBERG, J.C. & RUSH, B.F. (1966). An enzymatic spectrophotometric determination of pyruvic and lactic acid in blood. *Clin. Chem.*, **12**, 299.

SHRIEVE, D.C., DEEN, D.F. & HARRIS, J.W. (1983). Effects of extreme hypoxia on the growth and viability of EMT6/SF mouse tumor cells *in vitro*. *Cancer Res.*, **43**, 3521.

SCIANDRA, J.J., SUBJECK, J.R. & HUGHES, C.S. (1984). Induction of glucose-regulated proteins during anaerobic exposure and of heat-shock proteins after reoxygenation. *Proc. Natl Acad. Sci. USA*, **81**, 4843.

SMITH, E., STRATFORD, I.J. & ADAMS, G.E. (1980). Cytotoxicity of adriamycin on aerobic and hypoxic Chinese hamster V79 cells *in vitro*. *Br. J. Cancer*, **41**, 560.

SUBJECK, J.R. & SHYY, T.T. (1986). Stress protein systems of mammalian cells. *Am. J. Physiol.*, **250**, 1.

SUBJECK, J.R., SHYY, T., SHEN, J. & JOHNSON, R.J. (1983). Association between the 110,000 mammalian heat shock protein and nucleoli. *J. Cell. Biol.*, **97**, 1389.

SUTHERLAND, R.M., EDDY, H.A., BAREHAM, B., REICH, K. & VANANTWERP, D. (1979). Resistance to adriamycin in multicellular spheroids. *Int. J. Radiat. Oncol. Biol. Phys.*, **5**, 1225.

SUTHERLAND, R.M., KENG, P., CONROY, P.J., MCDERMOTT, D., BAREHAM, B.J. & PASSALACQUA, W. (1982). In vitro hypoxic cytotoxicity of nitromidazoles: update and cell cycle phase specificity. *Int. J. Radiat. Oncol. Biol. Phys.*, **8**, 745.

TEICHER, B.A., LAZO, J.S. & SARTORELLI, A.C. (1981). Classification of antineoplastic agents by their selective toxicities toward oxygenated and hypoxic tumor cells. *Cancer Res.*, **41**, 73.

WELCH, W.J., GARRELS, J.I., THOMAS, G.P., LIN, J.J.-C. & GERAMISCO, J.R. (1983). Biochemical characterisation of the mammalian stress proteins and identification of two stress proteins as glucse- and $Ca^{2+}$ -ionophore-regulated proteins. *J. Biol. Chem.*, **258**, 7102.

WELCH, W.J. & FERAMISCO, J.R. (1985). Rapid purification of mammalian 70,000-dalton stress proteins: affinity of the proteins for nucleotoides. *Molec. Cell. Biol.*, **5**, 1229.

WILSON, R.E., KENG, P.C. & SUTHERLAND, R.M. (1989). Drug resistance in Chinese hamster ovary cells during recovery from severe hypoxia. *J. Natl Cancer Inst.*, **81**, 1235.

WILSON, R.E. & SUTHERLAND, R.M. (1989). Enhanced synthesis of specific proteins, RNA, and DNA caused by hypoxia and reoxygenation. *Int. J. Radiat. Oncol. Biol. Phys.*, **16**, 957.